# Supplementary material for: Whole Genome Sequencing of Aggregatibacter actinomycetemcomitans Cultured from Blood Stream Infections Reveals Three Major Phylogenetic Groups Including a Novel Lineage Expressing Serotype a Membrane O Polysaccharide
Source: Pathogens. 2019 Nov 22;8(4):256. doi: 10.3390/pathogens8040256 (PMC6963875; doi:10.3390/pathogens8040256)
Supplement: Supplementary file 1 [file pathogens-08-00256-s001.pdf]

Strain	St[a]	Clade[b]	Lineage[c]	Host characteristics	Origin	Accession No	Study
NCTC_9710^T =ATCC_33384^T=CCUG_13227^T=DSM_8324T= HK_443^T, type strain					Abscess, Denmark 1955	VSDU00000000	
PN_434	c	c	I	Bacteremia	Denmark 1999	VSDV00000000	This study
PN_565	c	c	I	Bacteremia	Denmark 1998	VSEE00000000	This study
PN_566	c	c	I	Bacteremia	Denmark 1992	VSEF00000000	This study
PN_628	c	c	I	Bacteremia	Denmark 2015	VSEI00000000	This study
PN_686	c	c	I	Bacteremia	Denmark 2011	VSEM00000000	This study
PN_694	c	c	I	Bacteremia	Denmark 2008	VSEP00000000	This study
PN_708	c	c	I	Bacteremia	Denmark 2012	VSEQ00000000	This study
PN_728	c	c	I	Bacteremia	Denmark 2012	VSER00000000	This study
PN_773	c	c	I	Bacteremia	Denmark 2013	VSEV00000000	This study
PN_796	c	c	I	Bacteremia	Denmark 2002	VSEW00000000	This study
HK1651	b	b	I	Localized aggressive periodontitis	Denmark 1995	CP007502.1	[30]
PN_435	b	b	I	Bacteremia	Denmark 2006	VSDW00000000	This study
PN_436	b	b	I	Bacteremia	Denmark 2008	VSDX00000000	This study
PN_438	b	b	I	Bacteremia	Denmark 2011	VSDZ00000000	This study
PN_439	b	b	I	Bacteremia	Denmark 2013	VSEA00000000	This study
PN_627	b	b	I	Bacteremia	Denmark 2013	VSEH00000000	This study
PN_647	b	b	I	Bacteremia	Denmark 2012	VSEJ00000000	This study
PN_648	b	b	I	Bacteremia	Denmark 2012	VSEK00000000	This study
PN_684	b	b	I	Bacteremia	Denmark 2010	VSEL00000000	This study
PN_687	b	b	I	Bacteremia	Denmark 2012	VSEN00000000	This study
PN_738	b	b	I	Bacteremia	Denmark 2015	VSES00000000	This study
PN_756	b	b	I	Bacteremia	Denmark 2013	VSEU00000000	This study
PN_437	a	a/d	II	Bacteremia	Denmark 2008	VSDY00000000	This study
PN_559	a	a/d	II	Bacteremia	Denmark 1994	VSEB00000000	This study
PN_563	a	a/d	II	Bacteremia	Denmark 2007	VSED00000000	This study
PN_567	a	a/d	II	Bacteremia	Denmark 2014	VSEG00000000	This study
PN_688	a	a/d	II	Bacteremia	Denmark 2012	VSEO00000000	This study
PN_740	a	a/d	II	Bacteremia	Denmark 2006	VSET00000000	This study
D7S-1	a	a/d	II	Generalized aggressive periodontitis	USA before 2002	CP003496.2	[22]
I63B	d	a/d	II	Periodontally healthy subject	USA before 2000	AEJL00000000	[31]
A160	e	e/f	II	Chronic periodontitis	Finland 1995	AJME00000000	[31]
D18P-1	f	e/f	II	Generalized aggressive periodontitis	USA before 2000	AEJO00000000	[31]
NUM4039	g	-	II	Chronic periodontitis	Japan before 2010	AP014520	[32]
PN_696	a	-	III	Bacteremia	Denmark 2010	CP043005	This study
HK_907	a	-	III	(HG1185, T. J. M. van Steenbergen)	Holland before 1994	CP043003	[8]
HK_973	nt	-	III	Subgingival plaque, periodontal disease	Denmark 1991	CP043004	[8]
HK_974	a	-	III	(JC291-6, Jan Carlsson)	Sweden before 1994	VSDS00000000	[8]
HK_1710	a	-	III	Unknown	Brazil before 2007	VSDR00000000	This study
K51	a	-	III	Periodontal pocket	Kenya 2016	VSDT00000000	This study
PN_561	e	e’	(outgroup)	Bacteremia	Denmark 2005	VSEC00000000	This study
SC1083	e	e’	(outgroup)	Presumably a periodontally diseased subject	USA before 2010	AEJM00000000.1	[13]

[a] Serotype, mostly investigated by PCR; [b] Clade according to Kittichotirat 2016; [c] Lineage according to this study

Table S2. Lineage-specific genes (annotated by PROKKA)

Lineage I; 22 study strains including the type, 1 reference sequence
Lineage-specific genes, N=37
Hypothetical: 23
Annotated: 14
Acetolactate synthase isozyme 2 large subunit
Chorismate pyruvate-lyase
Dephospho-CoA kinase
DNA ligase
DNA topoisomerase 1
GDP-mannose 4,6-dehydratase
Glutamate 5-kinase
L-threonine dehydratase biosynthetic IlvA
L-threonine dehydratase biosynthetic IlvA
Opacity-associated protein OapA
Putative racemase YgeA
Signal recognition particle receptor FtsY
Stringent starvation protein B
tRNA-Val(tac)

Lineage II; 6 study strains, 5 reference sequences
Lineage-specific genes, N=89
Hypothetical: 73
Annotated: 16
Antitoxin HigA
Cell division protein FtsZ
Chaperone protein YcdY
Chorismate pyruvate-lyase
Cold shock-like protein CspC
CRISPR pre-crRNA endoribonuclease Cas5d
Dephospho-CoA kinase
Endoribonuclease HigB
Histidine biosynthesis bifunctional protein HisIE
Membrane sensor protein UhpC
Multidrug resistance protein MdtA
NADH dehydrogenase
Ribosomal protein S12 methylthiotransferase RimO
Swarming motility protein SwrC
Tyrosine recombinase XerC
Tyrosine-specific transport protein

Lineage III; 6 study strains
Lineage-specific genes, N=81
Hypothetical: 61
Annotated: 20
5-formyltetrahydrofolate cyclo-ligase
Beta-galactosidase
Beta-hexosaminidase
Chorismate pyruvate-lyase
CRISPR-associated endoribonuclease Cas2
CRISPR-associated exonuclease Cas4/endonuclease Cas1 fusion
Dephospho-CoA kinase
Ferrichrome outer membrane transporter/phage receptor
Glutamate racemase
IMPACT family member YigZ
Putative fluoride ion transporter CrcB
putative protein
putative protein/HI_1462.1
putative tRNA/rRNA methyltransferase
putative tRNA/rRNA methyltransferase YfiF
Regulatory protein RecX
Rhomboid protease GlpG
Ribosome-associated ATPase
Sulfur acceptor protein CsdE
Thymidylate kinase
